# Mice Immunized with IgG Anti-Sheep Red Blood Cells (SRBC) Together With SRBC Have a Suppressed Anti-SRBC Antibody Response but Generate Germinal Centers and Anti-IgG Antibodies in Response to the Passively Administered IgG

**DOI:** 10.3389/fimmu.2017.00911

**Published:** 2017-08-02

**Authors:** Joakim J. E. Bergström, Birgitta Heyman

**Affiliations:** ^1^Department of Medical Biochemistry and Microbiology, Uppsala University, Uppsala, Sweden

**Keywords:** sheep erythrocytes, IgG-mediated immune suppression, rhesus prophylaxis, germinal center, rheumatoid factor

## Abstract

Antigen-specific IgG antibodies, passively administered together with large particulate antigens such as erythrocytes, can completely suppress the antigen-specific antibody response. The mechanism behind has been elusive. Herein, we made the surprising observation that mice immunized with IgG anti-sheep red blood cells (SRBC) and SRBC, in spite of a severely suppressed anti-SRBC response, have a strong germinal center (GC) response. This occurred regardless of whether the passively administered IgG was of the same allotype as that of the recipient or not. Six days after immunization, the GC size and the number of GC B cells were higher in mice immunized with SRBC alone than in mice immunized with IgG and SRBC, but at the other time points these parameters were similar. GCs in the IgG-groups had a slight shift toward dark zone B cells 6 days after immunization and toward light zone B cells 10 days after immunization. The proportions of T follicular helper cells (T_FH_) and T follicular regulatory cells (T_FR_) were similar in the two groups. Interestingly, mice immunized with allogeneic IgG anti-SRBC together with SRBC mounted a vigorous antibody response against the passively administered suppressive IgG. Thus, although their anti-SRBC response was almost completely suppressed, an antibody response against allogeneic, and probably also syngeneic, IgG developed. This most likely explains the development of GCs in the absence of an anti-SRBC antibody response.

## Introduction

Antibodies, passively administered together with their specific antigen, have the ability to modulate the specific antibody response. This phenomenon is known as antibody feedback regulation (1–3). Whether the antibodies cause up- or downregulation of the antibody response depends both on the antibody isotype and the type of antigen used. IgM enhances responses to large antigens, such as erythrocytes and keyhole limpet hemocyanin, in a complement dependent manner (4–6). IgG and IgE enhance responses to soluble protein antigens and are dependent on the interaction with complement- or Fc-receptors (7–12). IgG, passively administered together with erythrocytes, can completely suppress the erythrocyte-specific antibody response (13–16). This has been used in the clinic since the 1960s to prevent immunization of RhD^−^ mothers carrying RhD^+^ fetuses (17, 18). Since its implementation, RhD prophylaxis has dramatically decreased the incidence of hemolytic disease of the fetus and newborn (19).

Although IgG-mediated suppression of antibody responses has been studied for decades, no consensus as to the mechanism behind has been reached. Suppression works well in all tested wild-type mouse strains, including C57BL/6 (20, 21), and in mice lacking activating FcγRs (15, 20, 22), the neonatal FcR, FcRn (15), the inhibitory FcγRIIB (14, 15, 22), as well as complement receptors 1 and 2, C1q, or C3 (20). In spite of its ability to almost completely suppress antibody responses, IgG administered with sheep red blood cells (SRBC) has little or no effect on the priming of specific CD4^+^ T helper cells (15, 23, 24). IgG-mediated suppression is dose dependent (13, 15), and suppression affects a wide range of parameters associated with a humoral immune response: primary IgM and IgG responses (13–16, 20), antigen-specific germinal center (GC) B cells (21), extra-follicular antibody-secreting cells (21), long-lived plasma cells (21), and induction of immunological memory (21). Suppression is restricted to the antigen to which the IgG antibodies bind (15) and no skewed suppression of certain IgG isotypes has been reported (24) [reviewed in Ref. (1)]. Here, we show that mice immunized with IgG anti-SRBC together with SRBC develop GCs although their anti-SRBC antibody response is severely suppressed. The GCs have a near-normal dark zone (DZ)/light zone (LZ) polarization and normal proportions of T follicular helper cells (T_FH_) and T follicular regulatory cells (T_FR_). A significant production of anti-IgG antibodies was detected in animals immunized with IgG together with SRBC, but not in animals immunized with IgG or SRBC alone. These observations suggest that GCs develop in response to the Fc-regions of the passively administered (suppressive) IgG antibodies. They highlight the interesting situation where IgG antibodies, bound to erythrocytes, (i) block access of B cells to the SRBC epitopes, resulting in “suppression” of an anti-SRBC response, and (ii) form arrays on the SRBC allowing IgG-specific B cells to bind, resulting in anti-IgG responses.

## Materials and Methods

### Mice

BALB/c mice were from Bommice (Ry, Denmark) and C57BL/6BomTac mice (C57BL/6) from Taconic Bioscience, Inc. (Hudson, NY, USA). Mice were age and sex matched within each experiment (both males and females were used) and were bred and maintained in the animal facilities of the National Veterinary Institute (Uppsala, Sweden). This study was carried out in accordance with the recommendations of the Uppsala Animal Research Ethics Committee, and the protocol was approved by the Uppsala Animal Research Ethics Committee.

### Antibodies and Antigens Used for Immunizations

Polyclonal IgG^b^ anti-SRBC was prepared from hyperimmune C57BL/6 serum, and polyclonal IgG^a^ anti-SRBC was prepared from hyperimmune BALB/c serum. IgG was purified by affinity chromatography using a Protein A Sepharose column (Amersham Pharmacia Biotech, Uppsala, Sweden) (25). Isolated IgG anti-SRBC was dialyzed against PBS, sterile filtered and stored at −20°C until use. SRBC in sterile Alsever’s solution were purchased from Håtunalab AB (Håtunaholm, Sweden) and stored at 4°C until use. SRBC were washed three times in PBS prior to use.

### Immunization and Blood Sampling

Mice were immunized with 200 µl SRBC ± 200 µl IgG anti-SRBC, both in PBS, in one of their lateral tail veins. Ten micrograms of IgG anti-SRBC were administered 30 min prior to 5 × 10^6^ SRBC. Controls received 5 × 10^6^ SRBC alone, 10 µg IgG anti-SRBC alone, or were left unimmunized. Blood was collected from the ventral tail artery. Details regarding IgG-allotypes are given in figure legends.

### Enzyme-Linked Immunosorbent Assay (ELISA)

To distinguish SRBC-specific IgG, actively produced in the immunized mice, from the passively administered IgG anti-SRBC an allotype-specific protocol was used (20). Briefly, ELISA plates were coated with 100 µl 0.25% SRBC and blocked with 5% dry milk in PBS. Serum samples were added, followed by a 1:1 mixture of biotinylated anti-mouse IgG1^a^ (clone 10.9) and IgG2a^a^ (clone 8.3) (BD Pharmingen, San Jose, CA, USA). Plates were developed using alkaline phosphatase conjugated to streptavidin (BD Pharmingen) and *p*-nitrophenylphosphate as substrate (Sigma-Aldrich). Absorbance at 405 nm was measured and analyzed using SoftMax software (Molecular Devices, Sunnyvale, CA, USA). The results are given as OD_405nm_ values, and serum dilutions are chosen so that the highest values do not reach plateau levels.

To detect IgG^a^ specific for IgG^b^, 96-well plates (Sigma-Aldrich) were coated with 100 µl 50 µg/ml IgG^b^ anti-SRBC in PBS overnight at 4°C and blocked with 5% dry milk in PBS for 2 h in room temperature prior to use. The remaining steps were performed as for the allotype-specific SRBC ELISA described above.

### Flow Cytometry

Single cell suspensions were prepared from spleens as described (26). Cells were resuspended in FACS buffer (2% fetal bovine serum in PBS) and incubated with Fc-block (anti-CD16/32; BD Biosciences) for 10 min on ice. GC B cells and DZ/LZ polarization were evaluated by staining with anti-B220-Pacific blue (clone RA3-6B2), anti-CD95-PECy7 (clone Jo2), anti-CD38-Alexa fluor 647 (clone 90), anti-CD83-PE (clone Michel-19) (BD Biosciences, San Jose, CA, USA), and anti-CD86-Biotin (clone GL1) (BD Pharmingen) for 30 min at 4°C. After washing twice in FACS buffer, Streptavidin-FITC (eBioscience, San Diego, CA, USA) was added, and samples incubated for 30 min at 4°C. T_FH_/T_FR_ populations were analyzed by surface staining with anti-CD4-PECy5 (clone GK1.5), anti-CXCR5-PECy7 (clone SPRCL5), and anti-PD1-Biotin (clone J43) (eBioscience). CXCR5 staining was performed at 37°C for 30 min and CD4 and PD1 at 4°C for 30 min. After washing twice in FACS buffer, Streptavidin-FITC (eBioscience) was added, and samples incubated for 30 min at 4°C. Intracellular staining of Foxp3 was performed using the Foxp3-PE kit from eBioscience according to the manufacturer’s suggestions. Cells were resuspended in 300 µl FACS buffer, and data were acquired using an LSR Fortessa cytometer (BD Biosciences) at the BioVis platform, Uppsala, Sweden and analyzed using FlowJo software (Tree Star Inc., Ashland, OR, USA).

### Confocal Laser Scanning Microscopy

Spleen sections for confocal microscopy were prepared as described (21). GCs were visualized by staining the sections with anti-B220-Pacific Blue (clone RA3-6B2) (BD Biosciences), anti-CD169 (MOMA)-FITC (clone MOMA-1) (Bio-Rad antibodies, Raleigh, NC, USA), and biotinylated peanut agglutinin (PNA; Vector Laboratories, Burlingame, CA, USA) for 1 h in room temperature. After washing twice in PBS, PE-conjugated streptavidin was added, and the slides were incubated for 1 h in room temperature and washed twice before mounting with Fluoromount G (Southern Biotech, Birmingham, AL, USA). Tile-scan images of immunofluorescence of whole spleen sections were acquired with an LSM 700 confocal microscope (Carl Zeiss, Thornwood, NY, USA) using Zen 2009 software (Carl Zeiss). Images were processed and analyzed with ImageJ software (NIH, Bethesda, MD, USA).

### Statistical Analysis

Statistical differences between groups were determined by the two-tailed Student’s *t*-test. Statistical significance levels were set as follows: ns, *p* > 0.05; *p < 0.05; **p < 0.01; ***p < 0.001.

## Results

### Development of GCs but Suppression of SRBC-Specific Antibody Responses in Mice Immunized with IgG Anti-SRBC Together With SRBC

**Figure 1:**
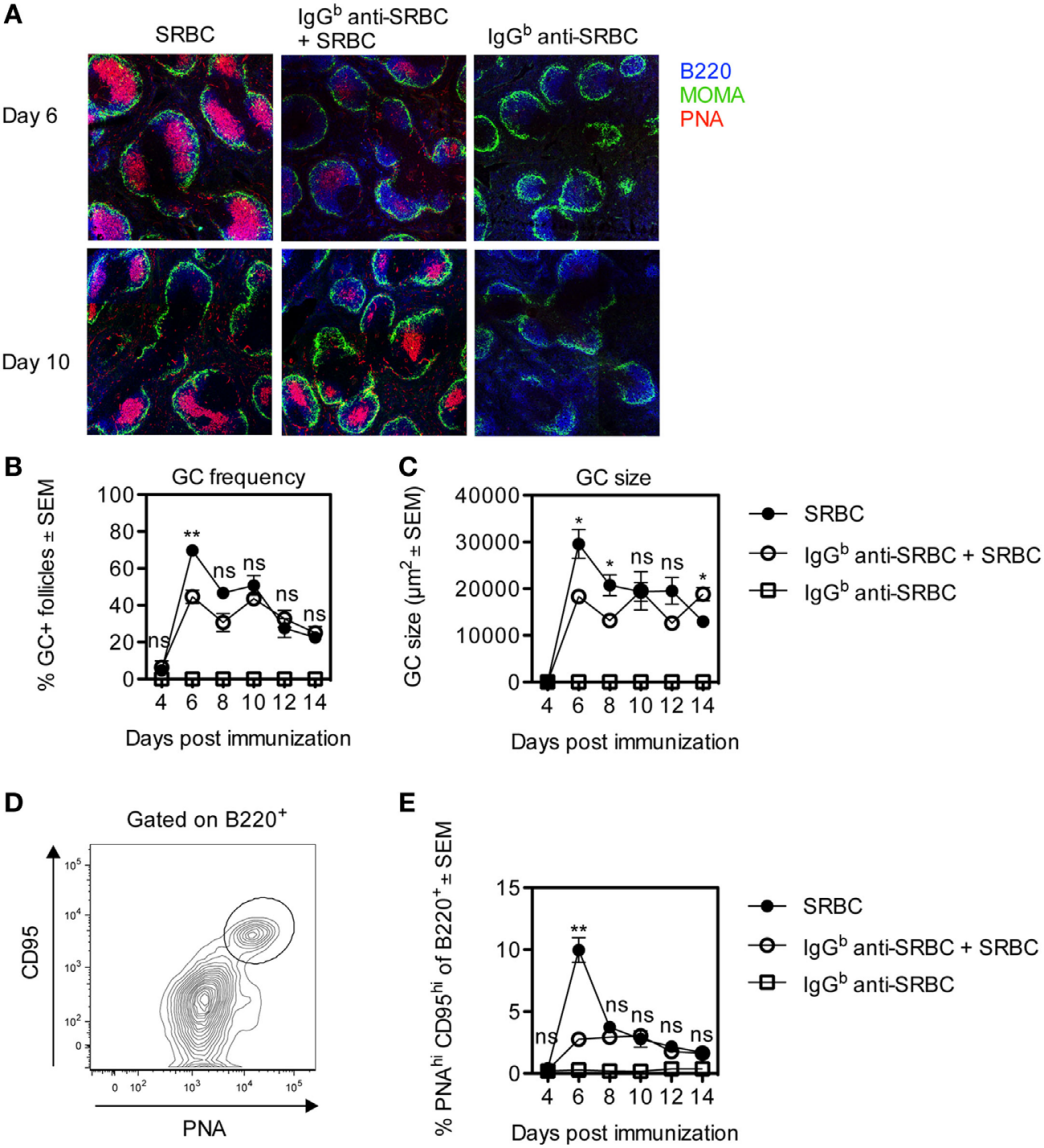
Development of germinal centers (GCs) in mice immunized with allogeneic IgG anti-sheep red blood cells (SRBC) together with SRBC. BALB/c mice were immunized with 10 µg IgG^b^ anti-SRBC 30 min prior to administration of 5 × 10^6^ SRBC (open circles). Controls were given 5 × 10^6^ SRBC alone (filled circles) or 10 µg IgG^b^ anti-SRBC alone (open squares). On days 4, 6, 8, 10, 12, and 14 after immunization, spleens were harvested. Half of each spleen was processed for analysis of GC B-cells by flow cytometry, and the other half for confocal laser scanning microscopy. **(A)** Visualization of PNA^+^ GCs in spleen sections 6 and 10 days after immunization: B220^+^ B-cells (blue), MOMA^+^ metallophilic macrophages (green), and PNA^+^ GCs (red). Image sizes are 1,500 µm × 1,500 µm. **(B)** Average percentage of follicles containing PNA^+^ GCs of total number of follicles. **(C)** Average size of PNA^+^ areas in the GCs in two non-consecutive spleen sections per mouse (10–40 GCs/section were measured) quantified as the area in square micrometers for each group. **(D)** GC B-cells gated as PNA^hi^CD95^hi^ of B220^+^ lymphocytes. **(E)** Percentage of PNA^hi^CD95^hi^ GC B-cells among B220^+^ lymphocytes. Data are representative of two independent experiments (*n* = 3/group) (ns, *p* > 0.05; *p < 0.05; **p < 0.01; ***p < 0.001).

Germinal center formation is important for affinity maturation and the generation of long-lived plasma cells and memory B-cells (27, 28). Specific IgG limits the amount of SRBC reaching the spleen (21, 24), and it seemed possible that IgG would also limit the induction of splenic GCs. To assess this, BALB/c mice were immunized with SRBC ± IgG^b^ anti-SRBC, and the GC response was followed for 4–14 days after immunization. Half of each spleen was analyzed by confocal laser scanning microscopy (Figures 1A–C). Six days after immunization, clearly visible PNA^+^ GCs had developed in both groups although at this time point the GC responses were higher in the groups immunized with SRBC alone than with IgG and SRBC (Figures 1A–C). Surprisingly, from day 8 to 10, the GC frequency (Figure 1B) and GC size (Figure 1C) were equally strong in both groups (Figures 1A–C). Immunization with IgG anti-SRBC alone did not induce GC formation (Figures 1A–C).

The other half of each spleen was analyzed by flow cytometry, and the gating strategy for CD95^hi^PNA^hi^ GC B-cells is shown (Figure 1D). After 6 days, the number of CD95^hi^PNA^hi^ GC B-cells in mice immunized with SRBC alone peaked and was fourfold to fivefold higher than in mice immunized with IgG anti-SRBC + SRBC (Figure 1E). Importantly, at all other time points GCs developed to the same extent in both groups (Figure 1E). No GC B-cells were detected in mice immunized with IgG anti-SRBC alone (Figure 1E). The same results were obtained when the percentage of GC B-cells was analyzed using other gating strategies, defining GC B-cells as CD95^hi^CD38^−^, GL7^hi^CD95^hi^, PNA^hi^CD95^hi^, or PNA^hi^GL7^hi^ cells (data not shown).

**Figure 2:**
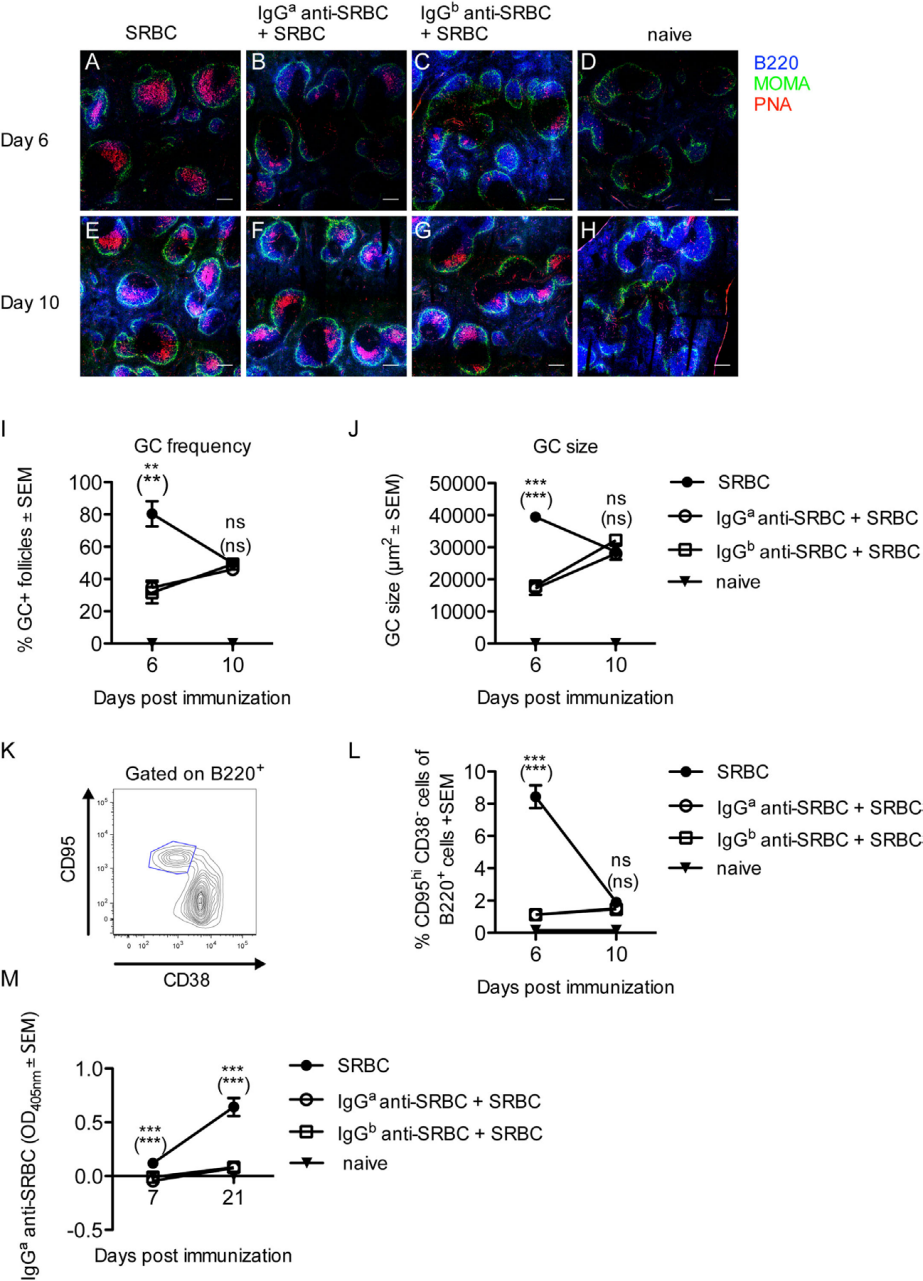
Development of germinal centers (GCs) but suppression of sheep red blood cells (SRBC)-specific antibody responses in mice immunized with syngeneic or allogeneic IgG anti-SRBC together with SRBC. BALB/c mice were immunized with 10 µg syngeneic IgG^a^ anti-SRBC (open circles) or 10 µg allogeneic IgG^b^ anti-SRBC (open squares) 30 min prior to immunization with 5 × 10^6^ SRBC. Controls were immunized with 5 × 10^6^ SRBC alone (filled circles) or left unimmunized (filled triangles). On days 6 and 10 after immunization, spleens were harvested. Half of each spleen was processed for analysis of GC B-cells by confocal laser scanning microscopy, and the other half for flow cytometry. **(A–H)** Representative 2,000 µm × 2,000 µm areas of tile scans of spleen sections. Visualization of GCs in the spleen: B220^+^ (blue), MOMA^+^ metallophilic macrophages (green), and PNA^+^ GCs (red) (*n* = 3/group). Scale bar is 200 µm. **(I)** Average percentage of follicles containing PNA^+^ GCs of total number of follicles. **(J)** Average size of PNA^+^ areas in the GCs in one spleen section per mouse (10–40 GCs/section were measured) quantified as the area in square micrometers for each group. **(K)** Representative contour plot for flow cytometric analysis of GC B-cells. **(L)** GC B-cells gated as CD95^hi^CD38^−^ of B220^+^ lymphocytes (*n* = 3/group). **(M)** The IgG^a^ anti-SRBC response was followed in mice from each group for 7–21 days after immunization (*n* = 5/group, *n* = 2 for negative controls). Sera diluted 1:625 were screened for IgG^a^ anti-SRBC in enzyme-linked immunosorbent assay. *p*-Values for comparisons of mice immunized with IgG^a^ anti-SRBC and SRBC versus SRBC alone are given without parentheses. Comparisons of mice immunized with IgG^b^ anti-SRBC and SRBC versus SRBC alone are given in parentheses. Data are representative of three **(A–J)**, at least four **(K,L)**, or two independent experiments/IgG allotype **(M)** (ns, *p* > 0.05; *p < 0.05; **p < 0.01; ***p < 0.001).

The administration of IgG^b^ anti-SRBC to BALB/c mice, with the Ig allotype a, constitutes an allogeneic situation (Figure 1). To investigate whether GC formation occurred to a similar extent in a syngeneic situation, BALB/c mice were immunized with SRBC alone or with SRBC together with either syngeneic IgG^a^ or allogeneic IgG^b^ anti-SRBC. The GC response was analyzed 6 and 10 days after immunization. After 6 days, PNA^+^ GCs had developed in all three groups (Figures 2A–C). At this time, the GC frequency (Figure 2I), the GC size (Figure 2J), and the number of GC B-cells (Figure 2L) were higher in mice immunized with SRBC alone than in mice immunized with either IgG^a^ or IgG^b^ together with SRBC. After 10 days, the GC response was similar in all three groups (Figures 2E–L).

To confirm that IgG indeed suppressed the anti-SRBC response, in spite of the strong GC reactions observed, additional mice from the groups analyzed for GC responses (Figures 2A–L) were bled and tested in ELISA. In BALB/c mice immunized with IgG^b^ anti-SRBC, the actively produced IgG^a^ anti-SRBC could be discriminated from the passively administered IgG^b^ anti-SRBC in an allotype-specific ELISA. In BALB/c mice immunized with IgG^a^ anti-SRBC, the ELISA detection antibodies will also detect the passively administered IgG. To compensate for this, a group of BALB/c mice was immunized with IgG^a^ anti-SRBC alone, and the OD_405nm_ values in their sera were subtracted from the OD_405nm_ values in mice immunized with IgG^a^ anti-SRBC together with SRBC. As expected, passively administered IgG severely suppressed the IgG anti-SRBC response, both in the allotype-specific ELISA (Figure 2M) and in an ELISA measuring total IgG anti-SRBC antibodies (Figure S1 in Supplementary Material).

In summary, GCs develop in mice where the primary SRBC-specific antibody response is severely suppressed either by syngeneic or allogeneic IgG anti-SRBC. Six days after immunization, the groups immunized with either IgG^a^ or IgG^b^ anti-SRBC and SRBC had a lower GC response than mice immunized with SRBC alone, but at other time points the GC responses were equally strong in all groups.

### IgG Induces a Minor Shift toward DZ B Cells on Day 6 and toward LZ B Cells on Day 10

**Figure 3:**
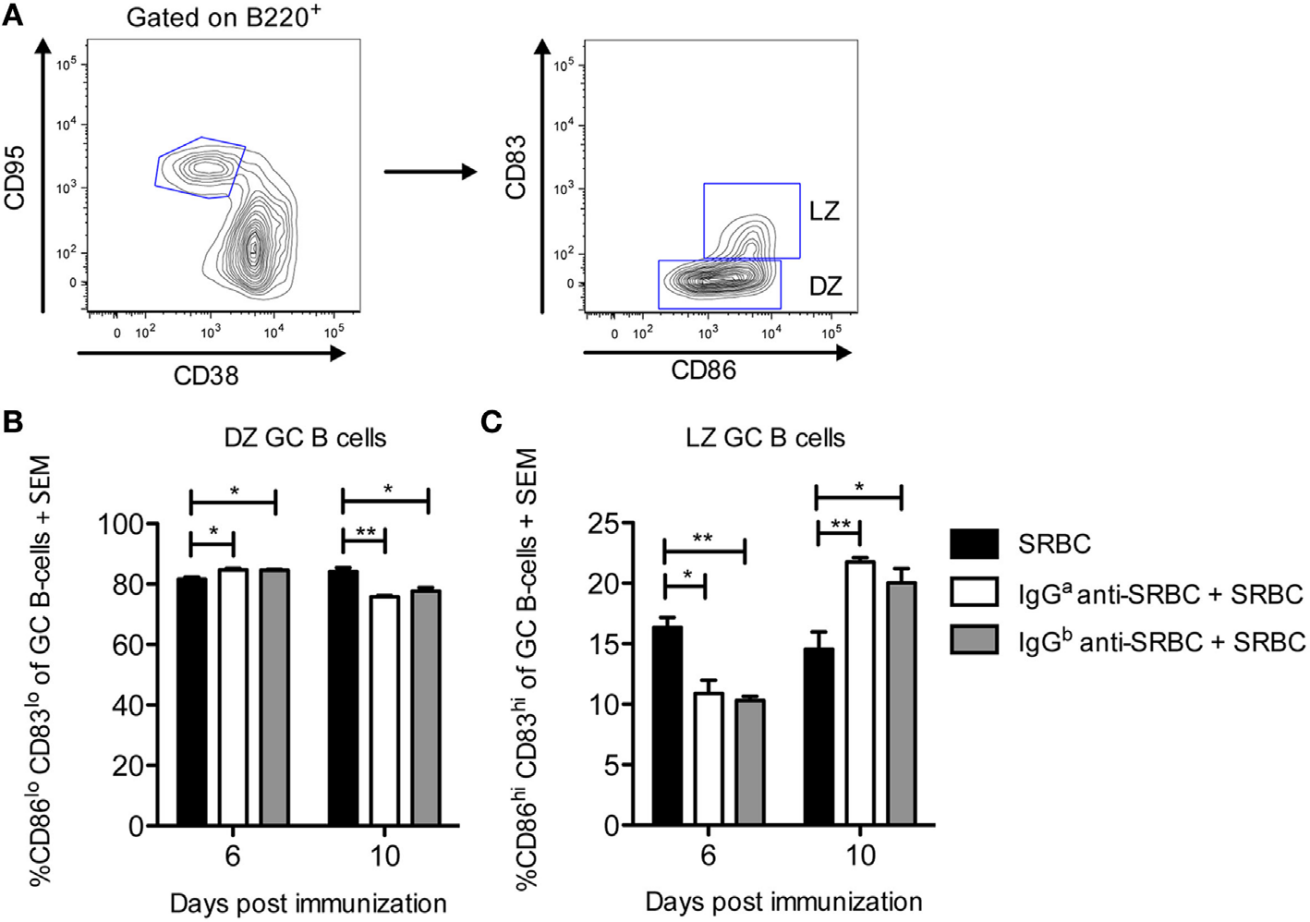
IgG induces a minor shift toward dark zone (DZ) B cells on day 6 and toward light zone (LZ) B cells on day 10. Splenocytes from the same spleens as in Figure 2 were used. On days 6 and 10 after immunization, spleens were harvested for analysis of DZ and LZ germinal center (GC) B-cells by flow cytometry. **(A)** Representative contour plot showing the gating strategy. **(B,C)** Quantification of flow cytometry data for groups immunized with 10 µg syngeneic IgG^a^ + 5 × 10^6^ sheep red blood cells (SRBC) (white bars), 10 µg allogeneic IgG^b^ + 5 × 10^6^ SRBC (gray bars), or 5 × 10^6^ SRBC alone (black bars) at indicated time points (*n* = 3/group). No GC B-cells were detected in unimmunized mice (not shown). Data represent % CD86^lo^CD83^lo^ DZ GC B-cells and % CD86^hi^CD83^hi^ LZ GC B-cells of B220^+^CD95^hi^CD38^−^ GC B-cells. Data are representative of three independent experiments/IgG allotype (ns, *p* > 0.05; *p < 0.05; **p < 0.01; ***p < 0.001).

Upon maturation, GCs polarize into two anatomically and functionally distinct zones, the DZ and the LZ (27, 28). The GC B-cells in these zones can be phenotypically distinguished: DZ B cells are characterized as CXCR4^lo^, CD86^lo^, CD83^lo^, and LZ B cells as CXCR4^hi^, CD86^hi^, and CD83^hi^ (29). To evaluate whether the passively administered IgG anti-SRBC affected the polarization of splenic GCs, spleens from the mice described in Figure 2 were analyzed. DZ and LZ CD95^hi^CD38^−^ GC B-cells were gated based on their relative surface expression of CD86 and CD83 as shown in (Figure 3A). Six days after immunization, IgG induced a small but clear shift from LZ to DZ phenotype, seen as a reduction in CD86^hi^CD83^hi^ LZ GC B cells (Figure 3C) and an increase in CD86^lo^CD83^lo^ DZ GC B cells (Figure 3B). However, 10 days after immunization, IgG induced a shift from DZ to LZ B cells, seen as an increase in CD86^hi^CD83^hi^ LZ GC B cells (Figure 3C), and a reduction in the CD86^lo^CD83^lo^ DZ GC B cells (Figure 3B). Thus, in IgG-suppressed mice, LZ GC B cells increased with time while DZ GC B cells decreased.

### IgG Does Not Change the Proportions of the T_FH_ and T_FR_ Populations

**Figure 4:**
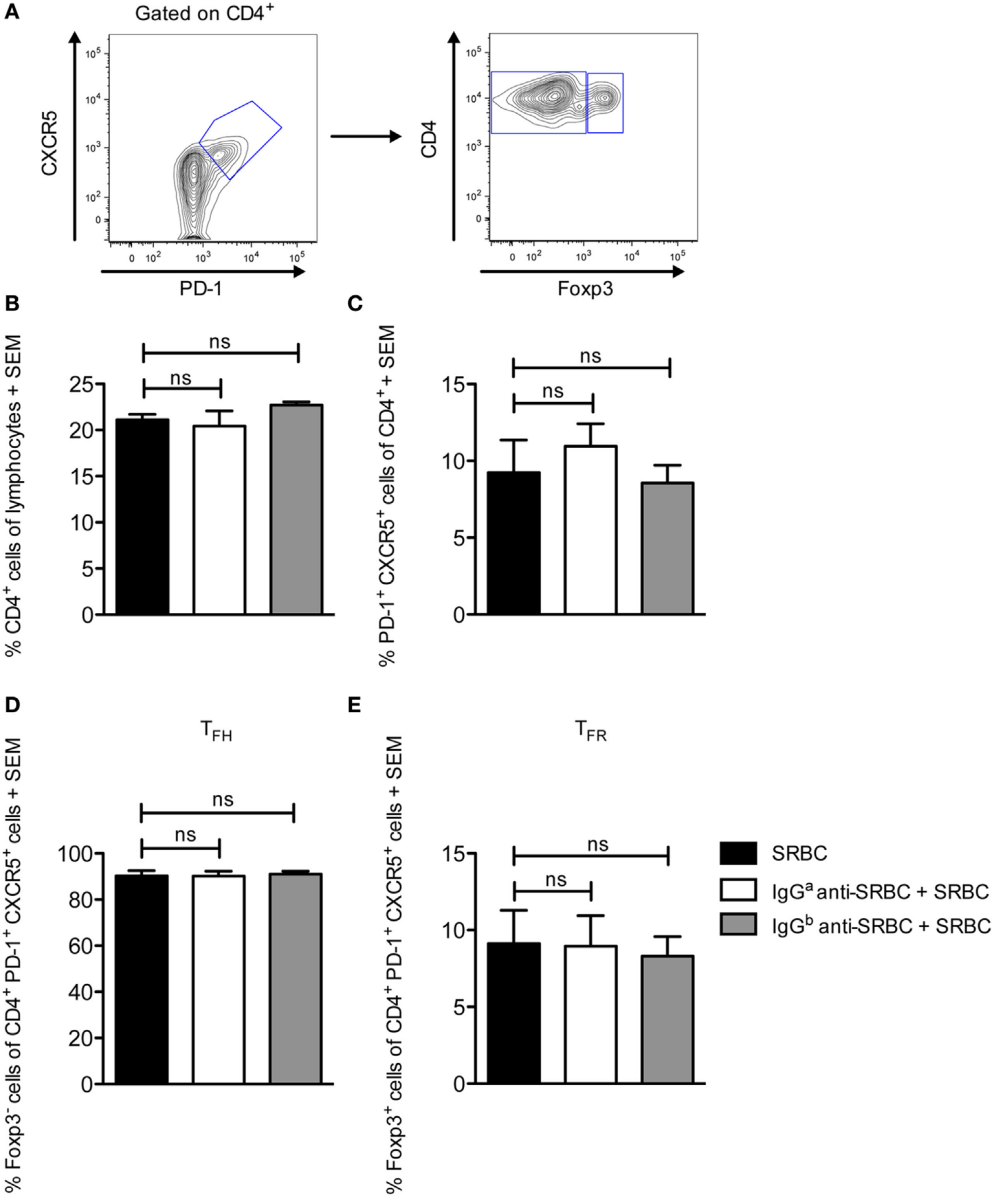
IgG does not change the proportions of the T_FH_ and T_FR_ populations. Splenocytes from the same spleens as in Figure 2 were used. On day 10 after immunization, spleens were harvested for analysis of T_FH_ and T_FR_ CD4^+^ cell populations by flow cytometry. **(A)** Representative contour plot showing the gating strategy. **(B–E)** Quantification of flow cytometry data for groups immunized with 10 µg syngeneic IgG^a^ + 5 × 10^6^ sheep red blood cells (SRBC) (white bars), 10 µg allogeneic IgG^b^ + 5 × 10^6^ SRBC (gray bars), and 5 × 10^6^ SRBC alone (black bars) (*n* = 3/group). Data represent **(B)** % CD4^+^ cells of lymphocytes, **(C)** % CXCR5^+^PD-1^+^, **(D)** % CXCR5^+^PD-1^+^Foxp3^−^ T_FH_, and **(E)** % CXCR5^+^PD-1^+^Foxp3^+^ T_FR_ of CD4^+^ lymphocytes. Data are representative of two independent experiments/IgG allotype (ns, *p* > 0.05; *p < 0.05; **p < 0.01; ***p < 0.001).

During the GC response, two effector subsets of CD4^+^ T cells play an essential role in regulating the response. T follicular helper cells (T_FH_) facilitate B cell selection and stimulate antibody production by providing limiting help to cognate GC B cells (27, 30–32). In contrast, T follicular regulatory cells (T_FR_) inhibit the GC reaction by suppressing cytokine production by T_FH_ as well as antibody production and class-switch recombination by B cells (33–35). How T_FR_ affect antibody responses is not completely understood, but the ratio between T_FH_ and T_FR_ seems to be of importance (36). Here, we sought to evaluate whether the proportion of these subsets was altered in IgG-suppressed mice, in spleens obtained from the mice described in Figure 2. All groups had comparable levels of CD4^+^ T cells (Figure 4B). In all three groups, CXCR5^+^PD-1^+^ cells comprised 7–13% of the CD4^+^ T cells (Figure 4C). Of these, slightly more than 90% were identified as T_FH_ (CD4^+^CXCR5^+^PD-1^+^Foxp3^−^) (Figures 4A,D), and slightly less than 10% as T_FR_ (CD4^+^CXCR5^+^PD-1^+^Foxp3^+^) (Figures 4A,E). In summary, administration of specific IgG together with SRBC does not alter the T_FH_ and T_FR_ proportions from those observed in mice immunized with SRBC alone.

### Immunization with IgG Anti-SRBC Together With SRBC Generates an Anti-IgG Response

**Figure 5:**
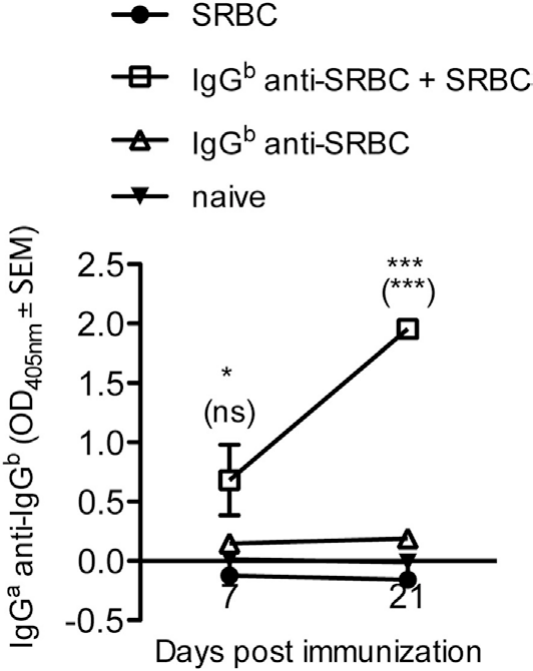
Immunization with IgG anti-SRBC together with sheep red blood cells (SRBC) generates an anti-IgG response. BALB/c mice were immunized with 10 µg allogeneic IgG^b^ anti-SRBC (open squares) 30 min prior to immunization with 5 × 10^6^ SRBC. Controls were immunized with 5 × 10^6^ SRBC alone (filled circles), 10 µg IgG^b^ anti-SRBC alone (open triangles), or left unimmunized (solid triangles). Sera were diluted 1:25 and screened for IgG^a^ anti-IgG^b^ by enzyme-linked immunosorbent assay. *p*-Values for comparisons of mice immunized with IgG^b^ anti-SRBC and SRBC versus SRBC alone are given without parentheses. Comparisons of mice immunized with IgG^b^ anti-SRBC and SRBC versus IgG^b^ anti-SRBC alone are given within parentheses. Data are representative of three independent experiments (*n* = 5/group, except for IgG^b^ anti-SRBC alone and naive groups where *n* = 2/group) (ns, *p* > 0.05; *p < 0.05; **p < 0.01; ***p < 0.001).

Administration of IgG anti-SRBC together with SRBC causes an almost complete suppression of the SRBC-specific antibody response (Figure 2M) but allows for a potent GC response (Figures 1 and 2). These apparently paradoxical observations raised the question of which antigen induces the GCs. One possibility is that the passively administered IgG antibodies, bound to the SRBC surface, are immunogenic. Indeed, BALB/c mice which had a suppressed SRBC response owing to immunization with IgG^b^ anti-SRBC together with SRBC (Figure 2M), had a potent IgG^a^ anti-IgG^b^ response (Figure 5). No anti-IgG^b^ response was seen in mice immunized with SRBC or IgG^b^ anti-SRBC alone (Figure 5). Thus, administration of allogeneic IgG anti-SRBC together with SRBC causes a potent antibody response against IgG (Figure 5) although no antibody response against SRBC is detected in the same sera (Figure 2M).

## Discussion

The presence of GCs in the absence of a detectable SRBC-specific antibody response in mice immunized with IgG anti-SRBC and SRBC was initially very puzzling. It is known that IgG suppresses primary IgM and IgG responses (13–16, 20), induction of memory and long-lived plasma cells as well as antigen-specific GC B cells and extra-follicular antibody-secreting cells (21). Therefore, it was unlikely that the development of GCs would be explained by a selective suppression only of GC-independent B cell differentiation steps while GC-dependent B cell differentiation was left untouched. A solution to the problem presented itself when a strong anti-IgG response was detected in mice immunized with allogeneic IgG anti-SRBC together with SRBC. This suggested that the GCs developed as a response to the passively administered IgG antibodies, which coat the SRBC surface, and not to the SRBC epitopes themselves. These findings agree well with a previous report showing that mice immunized with preformed complexes of allogeneic IgG anti-SRBC/SRBC or IgG anti-hen egg lysozyme (HEL)/SRBC-HEL, or with IgG anti-tetanus followed 24 h later by tetanus/diphtheria vaccine, produce anti-IgG antibodies in spite of a decreased antibody response to the classical antigen within the complex (37).

Interestingly, we observed equally efficient induction of GCs regardless of whether allogeneic or syngeneic IgG anti-SRBC was administered (Figure 2). However, whether an anti-IgG response occurred also in the syngeneic situation could not be analyzed. The ELISA plates would have to be coated with IgG^a^, and therefore the anti-IgG^a^ detection antibody would bind both directly to the coating and to any potential IgG^a^ anti-IgG^a^ from BALB/c sera binding to the coating. Hemagglutination was not a useful assay because the difference between direct and indirect titers was too small to allow reliable measurements of IgG^a^ anti-IgG^a^. Nevertheless, the great similarity between the GC parameters in mice immunized with either allogeneic or syngeneic IgG together with SRBC suggests that an antibody response against syngeneic IgG does indeed occur. Importantly, no anti-IgG was produced in mice immunized with IgG alone, suggesting that the presence of SRBC as a template for formation of the IgG arrays facilitates antibody responses to allogeneic IgG and possibly even breaks tolerance to syngeneic IgG. This is reminiscent of rheumatoid factors, autoantibodies specific for the IgG(Fc) region, which are frequently observed in rheumatoid arthritis.

Two differences between the GC development in mice immunized with IgG together with SRBC or with SRBC alone are apparent. First, the frequency and size of the GCs and the percentage of GC B cells are significantly higher in mice immunized with SRBC alone on day 6 (Figures 1 and 2). This is logical since two different antigens, SRBC or IgG, presumably induce the GCs: xenogeneic SRBC are most likely more immunogenic and cause a quicker GC development than allogeneic or syngeneic IgG. The other difference is the shift toward LZ B cells 10 days after immunization seen in mice immunized with IgG together with SRBC (Figure 3). In analogy with the reasoning above, this is probably explained by the fact that different antigens induce the GCs.

No consensus has been reached regarding the mechanism behind IgG-mediated suppression of antibody responses. Central inhibition of B-cell activation by co-crosslinking of the B-cell receptor with the inhibitory FcγRIIB, *via* IgG-antigen immune complexes, is unlikely because suppression works well in mice lacking FcγRIIB (14, 15, 20, 22). Involvement of activating FcγRs or complement is also unlikely. Suppression works well in FcRγ-chain deficient mice, which lack all activating FcγRs (15, 20, 22), as well as in mice lacking C1q, C3, or complement receptors 1 and 2 (21). Unexpectedly, a recent publication indicates that IgG-mediated suppression does not work in double knockout mice, lacking both C3 and FcRγ-chains, but works well in each single knockout (38). However, it remains to be elucidated how an IgG antibody response could be generated in the absence of C3 since it is has previously been established that IgG responses are severely impaired in the absence of C3, as well as C1q, C2, and C4, and complement receptors 1 and 2 [reviewed in Ref. (39, 40)].

Rapid clearance of IgG-RBC immune complexes has been discussed as a possible mechanism behind experimental IgG-mediated suppression and RhD prophylaxis. However, clinical trials with monoclonal IgG anti-RhD (41, 42) or with monoclonal IgG antibodies in murine experimental systems (16) do not show any correlation between clearance and suppression. Moreover, IgG efficiently suppressed the anti-SRBC response in FcRγ knockout mice although clearance of IgG–SRBC was severely impaired (21). The generation of an anti-IgG response in mice immunized with IgG anti-SRBC together with SRBC [Figure 5 and Ref. (37)] is hard to reconcile with clearance of the IgG–SRBC complexes as an explanation for IgG-mediated suppression: if clearance were important for this phenomenon, the antibody response to both SRBC and IgG would have been prevented.

From the reasoning above follows that many observations argue against Fc-dependent functions or clearance as explanations for IgG-mediated immune suppression. Another hypothesis that has been discussed is epitope masking. In addition to the independence of Fc-mediated functions demonstrated in knockout models, many other findings are compatible with this hypothesis. For example, suppression of IgG responses is epitope-specific (21), and F(ab′)_2_ fragments (15, 22, 43, 44) and IgE (15, 44) can suppress. Data from the present study also support epitope masking. The most straightforward explanation for why mice immunized with IgG anti-SRBC and SRBC do not respond to SRBC, although they generate GCs and produce anti-IgG antibodies, is that IgG binds to SRBC epitopes and “hides” them from SRBC-specific B cells. IgG anti-SRBC on the SRBC surface probably forms high density arrays because (i) there will be IgG molecules recognizing most of the different surface structures since polyclonal IgG anti-SRBC were administered, and (ii) previous calculations (15) show that 10 µg of IgG would be more than sufficient to cover the surface of 5 × 10^6^ SRBC (which are the doses used herein). Therefore, the surface-bound IgG molecules would be accessible to IgG-specific B cells while SRBC-specific B cells would be hindered from reaching the SRBC surface by these IgG molecules forming a dense layer on the cell surface.

Should epitope masking take place, B-cell epitopes would be inaccessible to specific B cells but the IgG–SRBC complexes would still be endocytosed and presented to CD4^+^ T helper cells. In line with this, IgG-mediated suppression of antibody responses is not paralleled by a suppression of specific T-cell responses (15, 23, 24). The lack of a difference in T_FH_ responses demonstrated here (Figure 4) is compatible with this, although we were not able to analyze antigen-specific T_FH_. The unperturbed numbers of T_FR_ in mice immunized with IgG and antigen (Figure 4) suggest that the suppressed anti-SRBC response is not due to T_FR_-induced immune suppression although a functional assay would be necessary to formally exclude this option.

In summary, the observations presented herein support the idea that IgG-mediated suppression of erythrocyte responses is caused by epitope masking. Moreover, they show that allogeneic, and most likely also syngeneic, IgG can induce antibody responses provided the IgG is presented to the immune system in a suitable way.

## Ethics Statement

This study was carried out in accordance with the recommendations of the Uppsala Animal Research Ethics Committee, and the protocol was approved by the Uppsala Animal Research Ethics Committee.

## Author Contributions

JB designed and performed experiments and wrote the manuscript. BH designed experiments and wrote the manuscript.

## Conflict of Interest Statement

The authors declare that the research was conducted in the absence of any commercial or financial relationships that could be construed as a potential conflict of interest.

## Abbreviations

C1q, complement factor 1q; C2, complement factor 2; C3, complement factor 3; C4, complement factor 4; DZ, dark zone; ELISA, enzyme-linked immunosorbent assay; FcγR, Fc gamma receptor; FcRγ, Fc receptor gamma chain; FcRn, the neonatal Fc receptor; GC, germinal center; HEL, hen egg lysozyme; LZ, light zone; RhD, rhesus D antigen; SRBC, sheep red blood cells; T_FH_, T follicular helper cells; T_FR_, T follicular regulatory cells.
