# Supplementary material for: Mice Immunized with IgG Anti-Sheep Red Blood Cells (SRBC) Together With SRBC Have a Suppressed Anti-SRBC Antibody Response but Generate Germinal Centers and Anti-IgG Antibodies in Response to the Passively Administered IgG
Source: Front Immunol. 2017 Aug 2;8:911. doi: 10.3389/fimmu.2017.00911 (PMC5539184; doi:10.3389/fimmu.2017.00911)
Supplement: Supplementary file 1 [file Image_1.PDF]

Supplementary Material

Mice Immunized with IgG anti-SRBC and SRBC have a Suppressed anti-SRBC Response but Generate Germinal Centers and anti-IgG Antibodies

Joakim J.E. Bergström[1] and Birgitta Heyman*[1)]
1) Department of Medical Biochemistry and Microbiology, Uppsala University, Uppsala, Sweden
*) Corresponding author: Birgitta Heyman, birgitta.heyman@imbim.uu.se

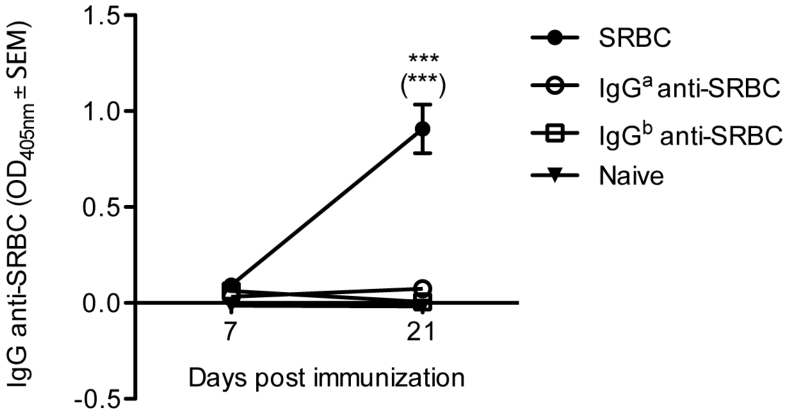

Supplemental Figure 1. Suppression of the total IgG anti-SRBC response.
The IgG anti-SRBC response in the same mice tested in Figure 2M was followed for 7-21 days after immunization (n=5/group, n=2 for negative controls). Sera diluted 1:625 were screened for IgG anti-SRBC in ELISA. p-values for comparisons of mice immunized with IgG^a anti-SRBC and SRBC versus SRBC alone are given without parentheses. Comparisons of mice immunized with IgG^b anti-SRBC and SRBC versus SRBC alone are given in parentheses. ns, $p > 0.05$; *, $p < 0.05$; **, $p < 0.01$; ***, $p < 0.001$.
